# Late Vitamin K Deficiency Bleeding in Infants Whose Parents Declined Vitamin K Prophylaxis — Tennessee, 2013

**Published:** 2013-11-15

**Authors:** Michael Warren, Angela Miller, Julie Traylor, Robert Sidonio, Anna Morad, Alyson Goodman, Deborah Christensen, Althea Grant, Erika Odom, Ekwutosi Okoroh, Joshua Clayton, Matthew Maenner, Lauren Marcewicz

**Affiliations:** Tennessee Dept of Health; Vanderbilt Univ; Div of Developmental Disabilities and Birth Defects; Div of Blood Disorders, National Center on Birth Defects and Developmental Disabilities; EIS officers, CDC

Vitamin K deficiency bleeding (VKDB) is a coagulopathy that develops in infants who do not have sufficient vitamin K stores to support production of clotting factors. In adults, vitamin K is absorbed from food and from vitamin K synthesized by gut bacteria. However, placental transfer in humans is limited; cord blood and infant liver reserve levels of vitamin K are substantially below adult levels (1,2). As a result, infants are predisposed to develop VKDB, which is classified as early, classic, and late, according to when it presents.* In the United States, administration of intramuscular vitamin K at birth to prevent all forms of VKDB has been standard practice since first recommended by the American Academy of Pediatrics in 1961 (3). Without this prophylaxis, incidence of early and classical VKDB ranges from 0.25% to 1.7% of births; incidence of late VKDB ranges from 4.4 to 7.2 per 100,000 infants (1–3). The relative risk for developing late VKDB has been estimated at 81 times greater among infants who do not receive intramuscular vitamin K than in infants who do receive it (4).

During February–September 2013, four confirmed cases of late vitamin K deficient bleeding were diagnosed at a children’s hospital in Nashville, Tennessee. The four infants had laboratory-confirmed coagulopathy, defined as elevation of prothrombin time (PT) greater than or equal to four times the laboratory limit of normal, correctable by vitamin K administration, and symptomatic bleeding. Three of the infants were born at major area hospitals, and one was born at home. The infants all had been healthy and developing normally until experiencing sudden symptomatic bleeding at age 6–15 weeks. Three of the infants had diffuse intracranial hemorrhage, and the fourth had gastrointestinal bleeding. Additionally, asymptomatic laboratory-confirmed coagulopathy was identified in the twin of one of the patients. In each case, parents had declined intramuscular vitamin K administration at birth. The Tennessee Department of Health initiated a public health investigation of this cluster and requested assistance from CDC.

All four of the infants survived. The infant with gastrointestinal bleeding recovered fully. The three with intracranial hemorrhage are being followed by neurologists; one has an apparent gross motor deficit. Although deficits have not yet been identified in the other infants, all are currently aged <1 year, and the neurodevelopmental impact of the hemorrhages might become apparent in the context of further development.

Preliminary queries of Tennessee hospital discharge data during 2007–2012 revealed no confirmed cases of late vitamin K deficiency bleeding, defined as an *International Classification of Diseases, Ninth Revision* (ICD-9) diagnosis code of either hemorrhagic disease of the newborn (776.0) or vitamin K deficiency (269.0), plus any codes for symptoms of bleeding, including intracranial or gastrointestinal hemorrhages, epistaxis, bruising, or hemothorax. During this period, 493,259 live births occurred in Tennessee. To assess the proportion of neonates who did not receive a vitamin K injection in 2013, records of a random sample of infants born during January–October 2013 at each of three Nashville area hospitals and at four major Tennessee nonhospital birthing centers were reviewed. At the Nashville hospital with the highest proportion of neonates not administered vitamin K, 3.4% of 3,080 infants discharged from the newborn nursery received no vitamin K injection. In contrast, 28.0% of 218 neonates at birthing centers did not receive vitamin K. Case-finding efforts revealed no additional cases of late VKDB in Tennessee in 2013.

Parents of the four infants with VKDB were asked why they declined vitamin K prophylaxis for their neonate. Reasons included concern about an increased risk for leukemia when vitamin K is administered, an impression that the injection was unnecessary, and a desire to minimize the newborn’s exposure to “toxins.” Concern about increased risk for leukemia in those receiving the vitamin K injection was initially generated by a 1992 report associating vitamin K injection and childhood cancer (5). The finding of an association with either leukemia specifically or general childhood cancer has not been replicated in other studies, but concern persists (1–3). In all cases, parental knowledge about the risk for development of late VKDB was either incomplete or absent at the time of declining prophylaxis, with most parents learning about the possibility of late VKDB only after their infants developed the condition.

This investigation is ongoing. A case-control study is under way to assess whether any additional risk factors might contribute to the development of late VKDB in children who do not receive vitamin K at birth. Record review at two more Nashville hospitals and one more nonhospital birthing center is in progress, and a survey of all parents identified through these record reviews who declined vitamin K administration for their children is planned to better understand why some parents decline this safe and effective prophylaxis.

## Footnotes

* Early VKDB occurs within 24 hours of birth, is often severe, and is almost exclusively in infants whose mothers used vitamin K–inhibitory drugs, such as antiepileptics and isoniazid, during pregnancy (1,2). Classic VKDB occurs between 24 hours and 7 days of life, and occurs as a result of the natural decrease in infant vitamin K levels occurring after separation from the placental source and before feeding can adequately replenish stores. Presentation is variable, ranging from mild bruising to severe intracranial hemorrhage (1). Late VKDB occurs in infants aged 2–24 weeks. Risk factors include breastfeeding and the presence of malabsorptive diseases such as cystic fibrosis and cholestatic liver disease, which prevent adequate consumption of dietary vitamin K. Presentations often include severe intracranial or gastrointestinal hemorrhage (1).

## References

[1] Zipursky A (1999). Prevention of vitamin K deficiency bleeding in newborns. Br J Haematol.

[2] Sutor AH, Kries R, Cornelissen EAM, McNinch AW, Andrew M (1999). Vitamin K deficiency bleeding (VKDB) in infancy. Thromb Haemost.

[3] American Academy of Pediatrics, Vitamin K Ad Hoc Task Force (1993). Controversies concerning vitamin K and the newborn. Pediatrics.

[4] McNinch AW, Tripp JH (1991). Haemorrhagic disease of the newborn in the British Isles: two year prospective study. BMJ.

[5] Golding J, Greenwood R, Birmingham K, Mott M (1992). Childhood cancer, intramuscular vitamin K, and pethidine given during labour. BMJ.

